# Mortality in the City of Chicago during the Month of September

**Published:** 1866-11

**Authors:** 


### MORTALITY IN THE CITY OF CHICAGO DURING THE MONTH OF SEPTEMBER.

The following is from the Health Officer's Report:

*Number of Deaths.*—Under 5 years of age, 329; from 5 to 10 years, 48; from 10 to 20 years, 36; from 20 to 30 years, 77; from 30 to 40 years, 106; from 40 to 50 years, 68; from 50 to 60 years, 41; from 60 to 70 years, 22; from 70 to 80 years, 3; from 80 to 90 years, 3. Unknown, 6. Total, 739.

*Nativities.*—Chicago, 311; other parts of the United States, 108; Bohemia, 17; Canada, 9; Denmark, 2; England, 15; France, 4; Finland, 2; Germany, 122; Holland, 6; Ireland, 83; Italy, 1; Norway, 25; Poland, 3; Russia, 1; Sweden, 15; Scotland, 4; Switzerland, 1; New Brunswick, 1; Wales, 1; unknown, 8. Total, 739.

*Causes of Death.*—Accident, 14; Burned, 1; Childbed, 5; Cholera, 166; Cholera Morbus, 36; Cholera Infantum, 6; Congestion, 2; Consumption, 29; Convulsion, 20; Cramp, 6; Diarrhœa, 23; Dysentery, 13; Drowned, 7; Fever, 70; Killed, 3; Pneumonia, 2; Small-pox, 1; Stillborn, 6; Summer Complaints, 87; Suicide, 3; Other causes, 161. Total, 739.

### CASES OF CHOLERA.

*Nativities.*—United States, 33; Bohemia, 9; Canada, 2; Denmark, 2; England, 6; France, 2; Germany, 110; Holland, 1; Ireland, 66; Norway, 19; Poland, 1; Sweden, 5; Switzerland, 2; Spain, 1; Scotland, 1; Italy, 1; unknown, 11. Total, 272.

*Ages.*—Under 5 years, 15; from 5 to 10 years, 22; from 10 to 20 years, 15; from 20 to 30 years, 64; from 30 to 40 years, 74; from 40 to 50 years, 40; from 50 to 60 years, 23; from 60 to 70 years, 7; from 70 to 80 years, 1; unknown age, 11. Total, 272.

The following is the distribution of cases in the several Wards:

| | | | | | |
|---|---|---|---|---|---|
| First, | 20 | Seventh, | 21 | Thirteenth, | 3 |
| Second, | 21 | Eighth, | 8 | Fourteenth, | 35 |
| Third, | 19 | Ninth, | 5 | Fifteenth, | 20 |
| Fourth, | 16 | Tenth, | 5 | Sixteenth, | 14 |
| Fifth, | 12 | Eleventh, | 19 | Unknown, | 2 |
| Sixth, | 15 | Twelfth, | 37 | | |
| | | Total, | | | 272 |

| | | | |
|---|---|---|---|
| Number of Males, | 143 | Sex unreported, | 4 |
| Number of Females, | 125 | | |
| | | Total, | 272 |

Number of deaths ascertained during the month, . 166

